# Slow binocular reading during rapid serial visual presentation (RSVP) in children with amblyopia and the role of fixation instability^☆^

**DOI:** 10.1016/j.visres.2025.108684

**Published:** 2025-09-09

**Authors:** Dorsa Mir Norouzi, Norah M. Nyangau, Yi-Zhong Wang, Lori M. Dao, Cynthia L. Beauchamp, David R. Stager, Jeffrey S. Hunter, Krista R. Kelly

**Affiliations:** a Retina Foundation of the Southwest, Dallas, TX, United States; b ABC Eyes Pediatric Ophthalmology, PA, Dallas, TX, United States; c Pediatric Ophthalmology & Adult Strabismus, PA, Plano, TX, United States; d Heaton Eye Associates, Tyler, TX, United States; e School of Optometry and Vision Science, University of Waterloo, Waterloo, ON, Canada

**Keywords:** Amblyopia, Binocular reading, Rapid serial visual presentation (RSVP), Fixation instability, Ocular motor dysfunction

## Abstract

Children with amblyopia read slower than their peers during binocular viewing. Ocular motor dysfunction typical of amblyopia may cause slow reading. It is unclear whether this is due to fixation instability or increased forward saccades. We examined whether removing the requirement of inter-word saccades helps children with amblyopia read at a similar rate as controls using a rapid serial visual presentation (RSVP) task. We also assessed whether reading rate was related to fixation instability. Children with amblyopia (n = 32) and control (n = 30) children ages 8–12 years silently read sentences presented in RSVP (single word presentation at screen center) during binocular viewing. Exposure time per sentence changed with a 2 – down 1 – up staircase to obtain reading speed thresholds (log words/minute [WPM]). Eye movements were tracked to determine fellow eye (FE) and amblyopic eye (AE) fixation stability during RSVP reading. Children with amblyopia read slower than controls (2.75 ± 0.47 log WPM vs 3.06 ± 0.40 log WPM), and had increased AE fixation instability (0.21 ± 0.39 log deg^2^ vs −0.20 ± 0.18 log deg^2^) and increased FE fixation instability (−0.03 ± 0.34 log deg^2^ vs −0.20 ± 0.15 log deg^2^) during RSVP reading. Reading rate in amblyopic children with good FE stability (n = 11) did not differ from controls and was faster than those with poor FE stability (n = 21). Children with poor FE stability read slower than controls. Removing the need for inter-word saccades (i.e., RSVP reading) did not help children with amblyopia read at control speeds. Our data support FE fixation instability as a source of slow reading in amblyopia.

## 1. Introduction

Amblyopia (‘lazy eye’), the most common cause of poor vision in one eye in children, affects up to 4 % or approximately 1 – 2 children per classroom. (Birch, 2013) Amblyopia is caused by discordant binocular input during early childhood from pediatric eye conditions, most commonly strabismus (eye misalignment), anisometropia (unequal refractive error), or a combination of both. Even with treatment to correct the refractive error and eye misalignment, vision is still poor in the affected eye. Current treatment for amblyopia includes occlusion (e. g., patching) of the fellow eye (FE) to force use of the amblyopic eye (AE). However, patching is not always successful at restoring normal vision and residual deficits in AE visual acuity often persist that are significantly impacted by a heightened sensitivity to crowding (i.e., difficulty identifying targets when flanked by other items). (Falkenberg et al., 2007; Levi and Klein, 1985) Binocular dysfunction including reduced or absent stereoacuity, interocular suppression, and motion perception deficits are also common. (Birch & Kelly, 2023) In addition, amblyopia is associated with ocular motor dysfunction, including the inability to maintain a stable gaze on a target (fixation stability), abnormal saccade initiation and execution, and reduced vergence. (Shaikh et al., 2016; Kelly et al., 2018; Subramanian et al., 2013; González et al., 2012; Chung et al., 2015; Kenyon et al., 1980; Maxwell et al., 1995; Perdziak et al., 2014; Perdziak et al., 2016).

As amblyopia emerges early in life during a critical period of maturation, these visual deficits can impact the development of functional vision tasks such as reading. Research has found that children read slower with their AE compared to the FE or to controls, which is expected given the poor visual acuity and abnormal crowding in that eye. (Repka et al., 2008; Levi et al., 2007; Stifter et al., 2005; Stifter et al., 2005) Yet, even under binocular viewing conditions that are more indicative of everyday life reading, amblyopic children are still slower at silent reading for comprehension than their visually-normal peers, despite one eye having good vision. (Stifter et al., 2005; Stifter et al., 2005; Kelly et al., 2023; Kelly et al., 2015; Kelly et al., 2017) Slow binocular reading in children with amblyopia does not depend on the type of amblyopia present (strabismic or anisometropic) or on sensory factors (AE visual acuity or binocular status). (Kelly et al., 2023; Kelly et al., 2015; Kelly et al., 2017) In fact, slow reading occurs during FE reading in amblyopia compared to controls reading monocularly, pointing to a FE deficit when reading with both eyes. (Kelly et al., 2023; Kanonidou et al., 2010).

The ocular motor dysfunction typical of amblyopia has been posited as the source of slow reading. Reading relies heavily on eye movements, such as forward or regressive saccades to move through the text and fixations (pauses) for word recognition and decoding. (Booth and Weger, 2013; Rayner, 1986) In general, research shows that slower reading speeds are linked to longer fixation durations and a smaller visual span, which then leads to more forward saccades. (Rayner, 1986; Rayner, 1998; Kwon et al., 2007) In children with amblyopia, fixation durations when reading are similar to controls. (Kelly et al., 2015) However, reading in amblyopia is associated with more forward saccades as they read through the text. (Kelly et al., 2015; Kelly et al., 2017; Kanonidou et al., 2010; Kanonidou et al., 2014; Bhutada et al., 2022) and is indirectly related to FE fixation instability. (Kelly et al., 2017) Thus, it is likely that slow binocular reading in amblyopic children is in part due to associated ocular motor dysfunction. However, the specific nature of the ocular motor dysfunction impacting reading is unclear. Children with amblyopia may make more restrained, smaller inter-word saccades, having difficultly positioning their gaze on the optimal part of the word and requiring minor corrective eye movements. Alternatively, they may be experiencing fixation instability that shortens fixation duration and the time available for word recognition and decoding. Fixational eye movements are small, involuntary movements that can be exacerbated in amblyopia, with instability comprised of saccadic intrusions, drift, and fusion maldevelopment nystagmus (FMNS). (Shaikh et al., 2016; Subramanian et al., 2013; Chung et al., 2015; Birch et al., 2013; Ghasia and Tychsen, 2024) While worse in the AE, fixation instability is present in both the AE and FE, (Shaikh et al., 2016; Kelly et al., 2018; Subramanian et al., 2013) which may interfere with optimal eye movement patterns during reading.

The Rapid Serial Visual Presentation (RSVP) paradigm is a method of presenting words one at a time at the same location on a display in quick succession. (Chung, 2021) The main advantage of RSVP reading is that although the need for fixating is required, the need for inter-word saccades is eliminated as the task requires fixating on a stationary location as the words appear sequentially in this location. A previous study used RSVP reading in adults with amblyopia, albeit for investigating crowding in the AE, not for assessing reading speed during binocular viewing. (Levi et al., 2007) Other studies used RSVP to train reading in adults with central vision loss due to age-related macular degeneration and Stargardt’s disease, citing improvements of 23 % for reading acuity and in maximum reading speed by 11–53 %. (Chung, 2011, 2021; Tarita-Nistor, Brent, Steinbach, Markowitz, & González, 2014) In the present study, we simultaneously recorded eye movements during RSVP reading in children with amblyopia compared to controls. Our primary goal was to use an RSVP reading task to determine whether removing the need for inter-word saccades during binocular reading will allow children with amblyopia to read at a similar speed as controls. Our secondary goal was to determine whether fixation instability contributes to RSVP reading performance in children with amblyopia.

## 2. Methods

### 2.1. Ethics

This research was conducted at the Retina Foundation of the Southwest (Dallas, Texas). The research protocol observed the tenets of the Declaration of Helsinki, was approved by the Institutional Review Board of the University of Texas Southwestern Medical Center and conformed to the requirements of the United States Health Insurance Portability and Privacy Act. Informed consent was obtained from a parent or legal guardian and verbal assent (<10 years) or written assent (≥10 years) was obtained from the child.

### 2.2. Participants

A total of 62 children were enrolled (32 amblyopic, 30 control). Children 8 to 12 years of age who had completed grades 2 to 6 were enrolled in the study between June 2021 and November 2022. Children with unilateral amblyopia were referred to the Retina Foundation of the Southwest by pediatric ophthalmologists or optometrists in the Dallas-Fort Worth area. Amblyopia was defined as FE best-corrected visual acuity (BCVA) better than or equal to 0.1 logMAR (20/25), AE BCVA worse than or equal to 0.2 logMAR (20/32), an interocular difference of at least 0.2 logMAR (2 lines), and an etiology of anisometropia and/or strabismus. Strabismic children were initially diagnosed with esotropia but were aligned with surgery or spectacles at the time of testing to within 4 prism diopters at near. Children with exotropia were excluded because exotropia in childhood is usually intermittent and does not typically cause amblyopia. (Mohney and Huffaker, 2003) Esotropia in infancy and childhood tends to be more constant and is therefore more commonly associated with amblyopia. (Birch & Stager, 1985; Tarczy-Hornoch, Varma, & Cotter, 2011) An age-similar group of normal control children with no history of vision disorders who had normal visual acuity and Randot stereoacuity were also enrolled. None of the children were born preterm (<32 weeks’ postmenstrual age) or had coexisting ocular or systemic disease, congenital infections/malformations, or developmental delay. None of the children were dyslexic or enrolled in school reading intervention programs. English was the primary language for all children. All children were tested with their habitual spectacle correction if required.

### 2.3. Procedures

#### 2.3.1. Vision assessment

Prior to reading, monocular best-corrected visual acuity (BCVA) was obtained for each eye using the electronic Early Treatment Diabetic Retinopathy Study (e-ETDRS; M&S Technologies, Illinois, USA) system, scored in logMAR. (Cotter et al., 2003) Stereoacuity was measured with the Randot Preschool Stereoacuity and Stereo Butterfly Tests (Stereo Optical, Inc; Illinois, USA), scored in log arcsec (range 1.3 to 3.3 log arcsec, nil stereoacuity assigned a value of 4). (Birch et al., 2008) Extent of suppression scotoma was evaluated using the Worth 4-dot (Bernell, Inc; Indiana, USA) test at seven different distances, measured as the farthest distance that the child reported four lights, scored in log degrees. (Webber et al., 2020; Rosenbaum et al., 1999).

#### 2.3.2. RSVP reading

Testing took place in a well-lit room. Eye movements were recorded during the RSVP task using a 500 Hz high-speed video binocular eye tracker (EyeLink 1000; SR Research, Ontario, Canada). Prior to testing, the experimenter first showed the child a sentence on a piece of paper and asked them a ‘Yes’ or ‘No’ question to provide an example of the type of sentences they will read. Following this initial example, the child sat at a habitual reading distance of 40 cm from a computer monitor with their head stabilized using a head/chin rest. To avoid issues with calibrating children with fixation instability typical of amblyopia, a custom 5-point monocular calibration was performed on each eye while occluding the nonviewing eye using the EyeLink 1000 eye tracker.

**Fig. 1.**
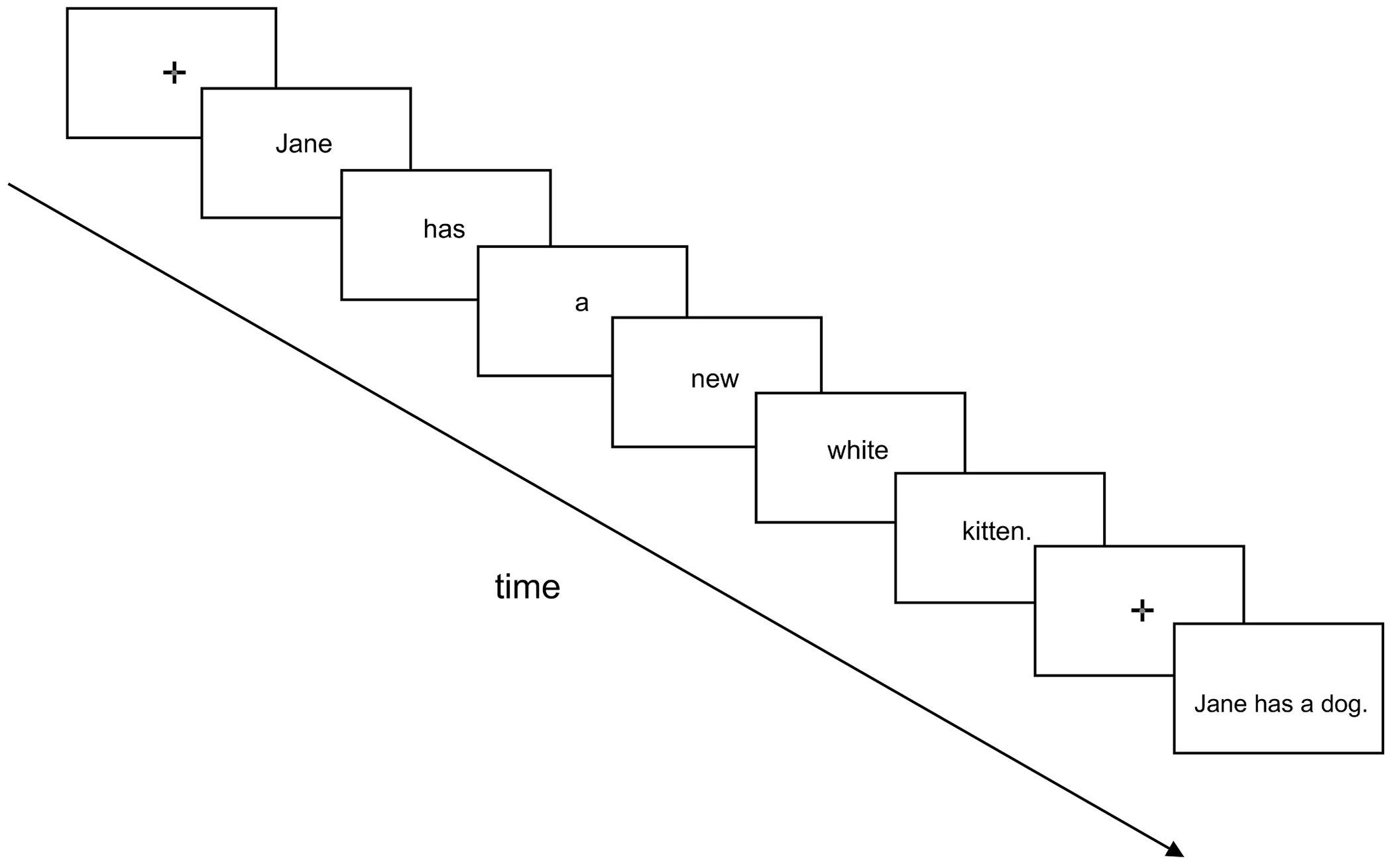
Example of an RSVP trial. The child was instructed to fixate the red dot in the center of the cross. The fixation cross disappeared, and a sentence was presented one word at a time with equal exposure time in rapid serial visual presentation (RSVP) in the center of the screen. A second fixation cross appeared at the end of the sentence and then a question was presented. The experimenter read the question to the child and the child responded ‘Yes’ or ‘No’ out loud to the experimenter.

Children were asked to silently read sentences presented in rapid serial visual presentation (RSVP; single word presentation at screen center; example in Fig. 1) with both eyes open. The sentences and questions were chosen from grade appropriate stories in the Readalyzer (Compeva AB, Stockholm, Sweden) book used in previous studies from our lab. (Kelly et al., 2023; Kelly et al., 2015; Kelly et al., 2017) The font type was SansSerif and font size corresponded to 20/160 (0.9 logMAR) for those who completed grades 2 – 3, and 20/100 (0.7 logMAR) for those who completed grades 4 – 6. The longest words for each grade were between 9 and 13 letters (grade 2 – 9 letters; grade 3 – 11 letters; grade 4 – 10 letters; grade 5 – 11 letters; grade 6 –13 letters).

Two practice trials were provided – one at 500 msec presentation time and one at 250 msec presentation time per word – to ensure the child understood the task. For each trial, participants were first presented with a black cross that had a small red dot in the center of the screen that they were instructed to fixate and to keep their eyes in this position during the sentence presentation. Each word of the sentence appeared one at a time at a constant exposure time, with the initial trial starting at 500 msec between each word. The participant was then asked a ‘Yes’ or ‘No’ question at the end of each sentence to verify comprehension and that they could read the sentence. The child let the experiment know their answer by saying it out loud. A descending adaptive 2 — down 1 — up staircase method was used to determine the child’s reading speed threshold. After two correct trials at a single exposure time (2 — down), the exposure time decreased by 25 msec for the next trial (i.e., faster presentation of the words). If the participant answered the question incorrectly (1 — up), the exposure time increased by 25 msec for the next trial (i.e., slower presentation of the words). The experiment was terminated once the participant had made six reversals or had completed all the trials (max of 35 to 41 trials, depending on grade level). A reading speed threshold was then calculated for each child based on the average of the last four reversals, converted to log words per minute (WPM). Each exposure time corresponds to a certain reading speed in WPM based on the formula WPM = 60,000/Exposure Time. For example, an exposure time of 500 msec per word equals 120 words WPM whereas an exposure time of 250 msec per word equals 240 WPM.

#### 2.3.3. Eye movement data processing

Eye movement data exported from the EyeLink Data Viewer were processed and visualized using a graphical user interface custom-built in MATLAB (Mathworks, Natick, MA). Horizontal and vertical monocular calibration values were applied to the eye position data for each eye. Only eye movements that occurred during fixation of the cross that initiated each trial and during the RSVP presentation were recorded; eye movements during the question period were removed. Blinks, artifacts, and large saccades (> 5°) detected by EyeLink were removed prior to analysis. Fixation instability was quantified during the RSVP task by calculating the bivariate contour ellipse area (BCEA; log deg^2^), an area within which 68 % of all the x-,y-coordinates of fixation points occurred, (Kelly et al., 2018; González et al., 2012) for the total duration of the experiment. Because participants were instructed to fixate on the cross and keep their eyes in this position as each RSVP trial appeared, we were able to calculate one total BCEA per participant that included horizontal and vertical eye positions for all experimental trials, with eye position data from practice trials and from the question period removed.

#### 2.3.4. Statistical analyses

Independent t-tests were conducted to determine group differences (amblyopia vs control) in reading rate (log WPM) and fixation instability for the AE (right eye for controls) and FE (left eye for controls). To assess the contribution of sensory factors in reading rate in the amblyopic group, we performed partial correlations with reading rate (log WPM) and AE BCVA, stereoacuity, and Worth 4-dot fusion while controlling for grade level completed. To determine the effect of etiology on RSVP reading, a Kruskal-Wallis H test was conducted to assess differences in reading rate among the different types of amblyopia (strabismic, anisometropic, combined mechanism). Lastly, children with amblyopia were binned into having good FE stability and poor FE stability based on the upper limit of the 95 % confidence interval of the control group, and a Kruskal-Wallis H test was conducted to determine group differences (good FE stability, poor FE stability, controls) in reading rate. Mann Whitney U tests were used to conduct posthoc pairwise comparisons for significant Kruskal-Wallis H tests.

## 3. Results

Group characteristics for the amblyopia and control groups are shown in Table 1. Groups did not differ in last grade completed (U = 409, p = 0.31) and no sex differences were found for log WPM for the amblyopic (p = 0.20) or control (p = 0.88) groups. Eye tracking data from 2 control children were not useable due to poor tracking. Eye tracking data from all amblyopic children were useable.

**Fig. 2.**
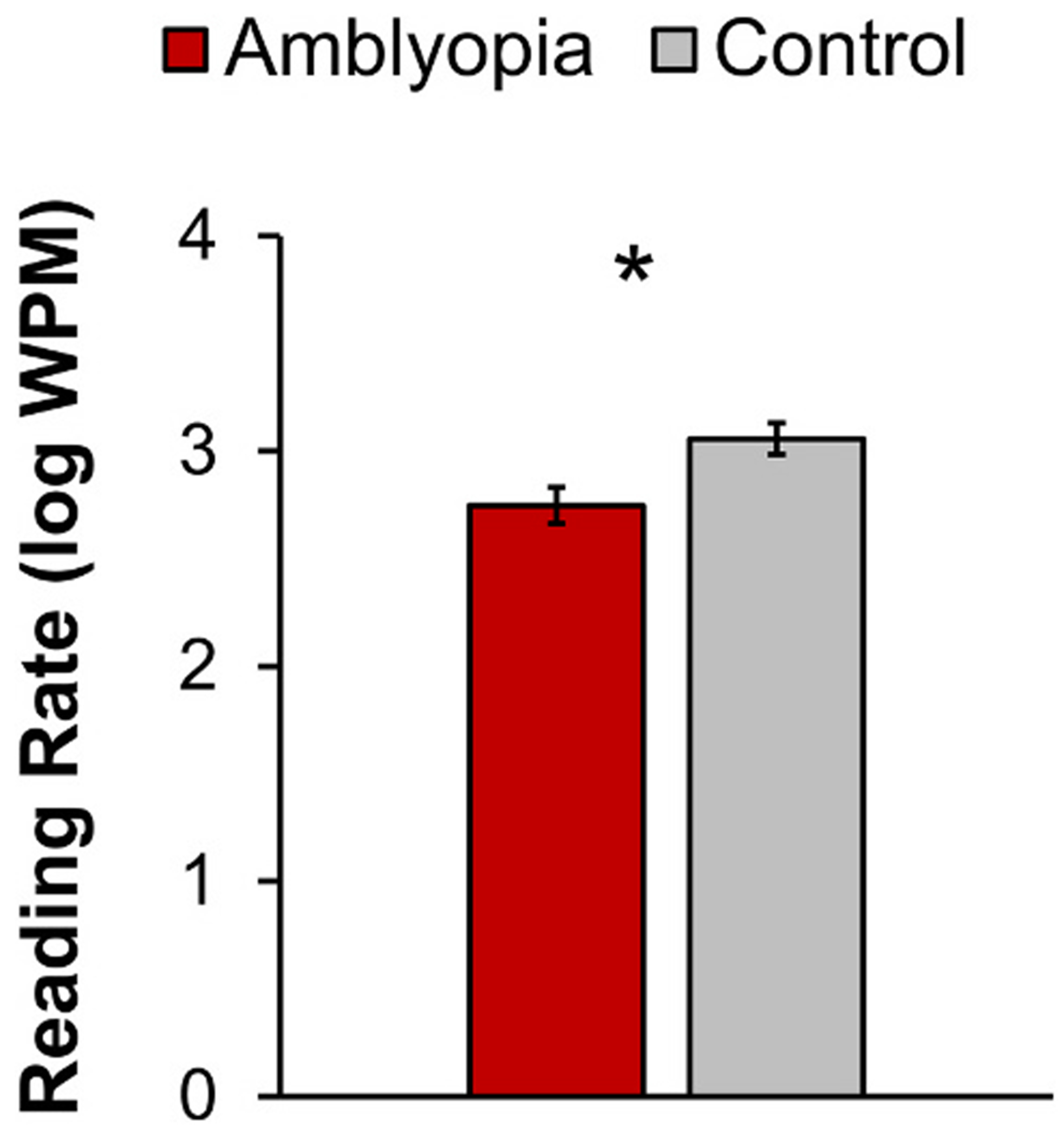
Reading rate. Bar graphs depicting mean reading rate in log words per minute (WPM) during binocular RSVP reading for children with amblyopia (red bar) compared to control children (grey bar). The amblyopia group read slower (i.e., had lower log WPM) than controls. Error bars represent ± standard error of the mean (SEM). *significant at p < 0.05. (For interpretation of the references to colour in this figure legend, the reader is referred to the web version of this article.)

Children with amblyopia read slower than controls during binocular RSVP reading (amblyopia, mean ± SD = 2.75 ± 0.47 log WPM vs control, 3.06 ± 0.40 log WPM, t_60_ = 2.57, p = 0.007, d = 0.70; Fig. 2). For the amblyopia group, reading rate was not significantly correlated with AE BCVA (r = 0.01, p = 0.98), stereoacuity (r = 0.12, p = 0.53), or Worth 4-dot fusion (r = 0.07, p = 0.72). There was no difference in last grade completed (H = 1.46, p = 0.48) or reading rate (H = 2.12, p = 0.35) between the different types of amblyopia.

**Fig. 3.**
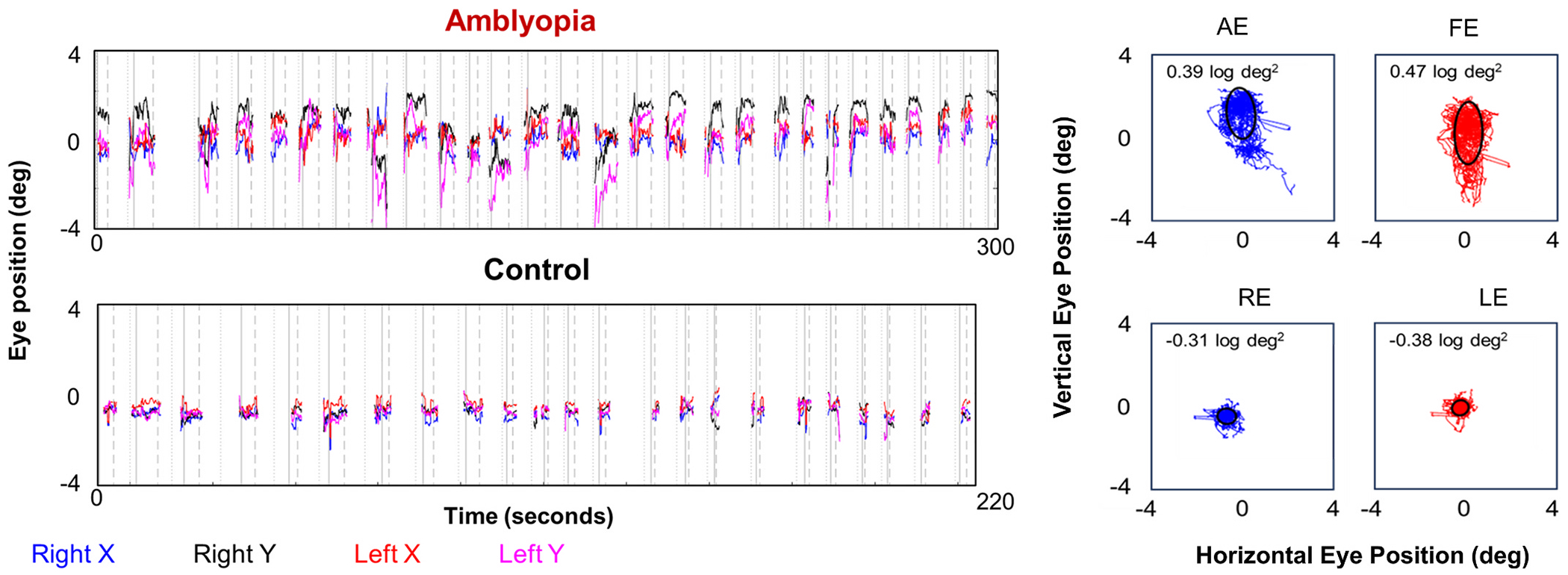
Example eye traces and BCEAs (log deg^2^) during RSVP reading for a child with amblyopia (top) and a control child (bottom). The amblyopic child had a slower reading rate than the control child (2.3 log WPM vs 3.2 log WPM). Small, dotted lines represent fixation target onset, solid lines represent onset of the RSVP sentence, dashed lines represent end of the RSVP sentence and start of the question period (eyes not tracked).

**Fig. 4.**
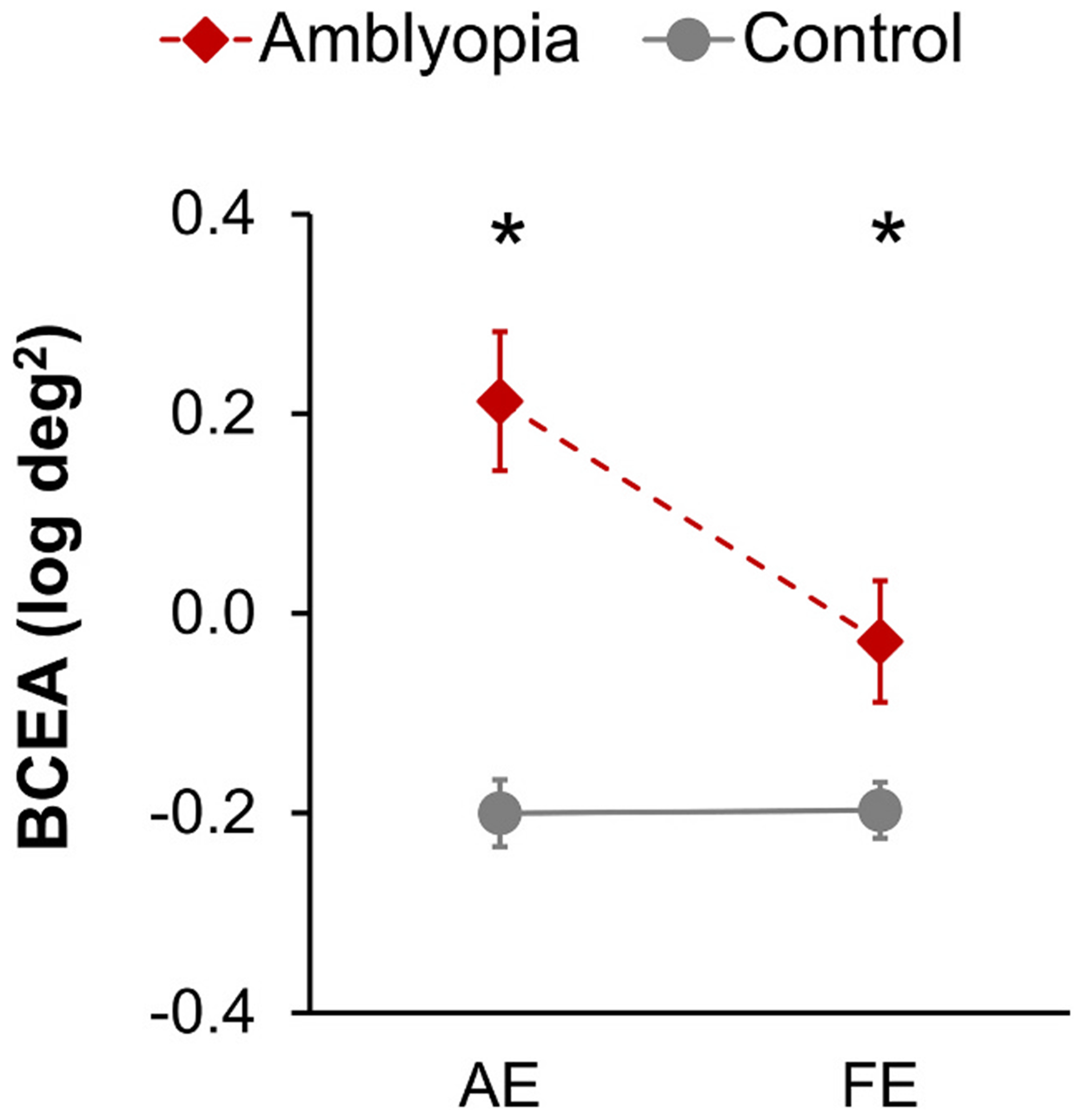
Fixation instability during RSVP reading. Line graph depicting mean fixation instability (BCEA: log deg^2^) for the amblyopic eye (AE) and fellow eye (FE) as children fixate in the center of the screen during RSVP reading. Children with amblyopia (red triangles) had larger fixation instability compared with controls (grey circles) in both the AE and FE during RSVP reading. Error bars represent ± standard error of the mean (SEM). *significant at p < 0.05. (For interpretation of the references to colour in this figure legend, the reader is referred to the web version of this article.)

During binocular RSVP reading, children with amblyopia had larger AE fixation instability compared to the control group’s right eye stability (amblyopia, 0.21 ± 0.39 log deg^2^ vs control, −0.20 ± 0.18 log deg^2^, t_58_ = 5.35, p < 0.001, d = 1.32) and larger FE instability compared to controls’ left eye stability (amblyopia, −0.03 ± 0.34 log deg^2^ vs control, −0.20 ± 0.15 log deg^2^, t_57_ = 2.52, p = 0.015, d = 0.62). There was no difference in fixation stability between the right and left eye for controls (right, −0.20 ± 0.18 log deg^2^ vs. left, −0.20 ± 0.15 log deg^2^, t_26_ = 0.61, p = 0.55, d = 0.12). See Fig. 3 for example eye traces during RSVP reading and the corresponding BCEAs, and Fig. 4 for group means.

**Fig. 5.**
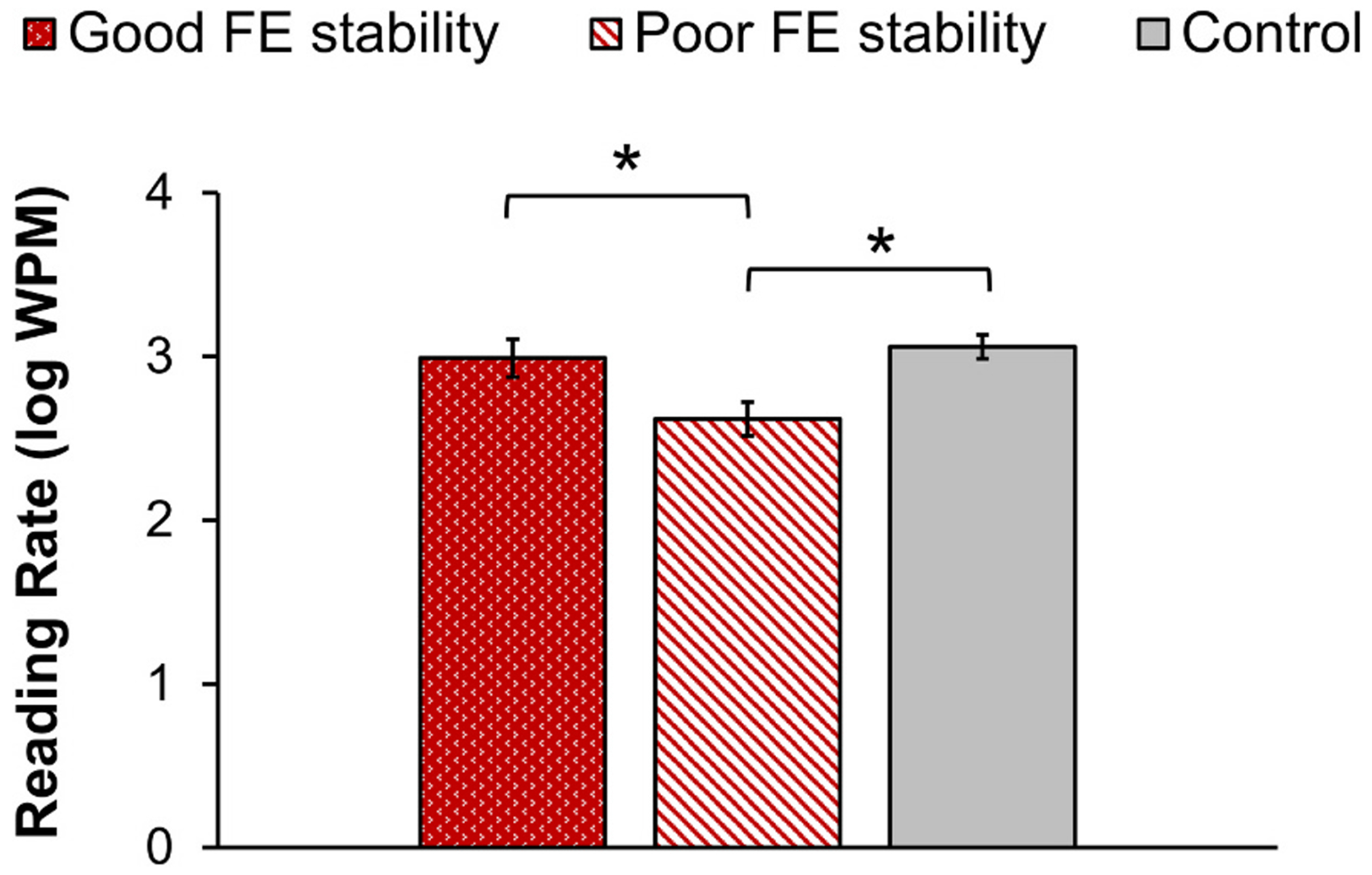
Reading rate and fixation instability. Bar graphs depicting mean reading rate in log words per minute (WPM) during binocular RSVP reading for amblyopic children with good fellow eye (FE) stability (red bars with white dots), amblyopic children with poor FE stability (white bar with red stripes) and control children (grey bar). Amblyopic children with good FE stability read at a similar rate as controls, while those with poor FE stability read slower (i.e., had lower log WPM) than controls and those with good FE stability. Error bars represent ± standard error of the mean (SEM). *significant at p < 0.05. (For interpretation of the references to colour in this figure legend, the reader is referred to the web version of this article.)

To determine the role of FE fixation stability in reading, amblyopic children were binned into two groups based on the upper limit of the control group’s 95 % confidence interval for fixation stability of the left eye (range = −0.25 to −0.13); *good FE stability* (BCEA ≤ −0.13 log deg^2^, n = 11) and *poor FE stability* (BCEA > −0.13 log deg^2^, n = 21). Groups did not differ in last grade completed (H = 2.57, p = 0.28). There was a significant main effect of group (H = 11.13, p = 0.004; Fig. 5). Posthoc pairwise comparisons show that reading rate in amblyopic children with good FE stability did not differ from controls (good FE stability, 2.99 ± 0.39 log WPM vs controls 3.06 ± 0.40 log WPM, p = 0.65), while amblyopic children with poor FE stability read slower than controls (poor FE stability, 2.61 ± 0.47 log WPM vs controls, 3.06 ± 0.40 log WPM, p = 0.002) and those with good FE stability (p = 0.022). The role of AE fixation stability in reading was unable to be assessed as only 6/32 (19 %) children in the amblyopia group had good AE fixation stability. Half (3/6) had reading rates as fast as controls, and among the fast readers, 2/3 had good FE stability.

## 4. Discussion

We investigated whether eliminating the need for inter-word saccades using RSVP allows children with amblyopia to read at rates comparable to controls. We also examined the relationship between reading rate and fixation instability. Children with amblyopia read slower than controls during binocular RSVP reading, despite having normal visual acuity in the FE. Further, sensory factors such as AE BCVA, stereoacuity, and fusion/suppression were not related to RSVP reading rate, nor was there any difference between strabismic or anisometropic amblyopia types, consistent with previous studies. (Stifter et al., 2005; Kelly et al., 2023; Kelly et al., 2015; Kelly et al., 2017; Kanonidou et al., 2014; Buczkowska and Kowiak, 2017) Our finding that removing the requirement for inter-word saccades using an RSVP reading task did not help children with amblyopia read as fast as controls may appear to contrast with studies showing increased saccades during reading in children with amblyopia. (Kelly et al., 2015; Kelly et al., 2017; Kanonidou et al., 2010; Kanonidou et al., 2014; Bhutada et al., 2022) However, increased saccades may still be a contributing factor to slow reading when eye movements are required, as in paragraph reading. (Kelly et al., 2023; Kelly et al., 2015; Kelly et al., 2017) In addition, the increase in saccades in previous studies may be related to fixation instability typical of amblyopia. (Shaikh et al., 2016; Kelly et al., 2018).

In addition to a problem with moving the eyes between words, slow reading in amblyopia may be due to FE fixation instability. Even when fixing a target, the eyes are constantly in motion, helping to prevent visual fading from retinal neural adaptation. However, when these fixational eye movements are excessive (i.e., saccadic intrusions, increased drift, FMNS), fixation instability occurs. Fixation instability is present in strabismic and anisometropic amblyopia in both the AE and FE. (Shaikh et al., 2016; Kelly et al., 2018; Ghasia et al., 2018) Thus, although visual acuity is fine in the FE, fixation stability is not normal in the FE during reading, which is likely the eye being used to read due to suppression of the AE. In our study, children with poor FE fixation instability had slower RSVP reading rates compared to controls and compared to amblyopic children with good FE fixation instability. This finding is also supported by a study showing an indirect relationship of FE fixation instability and slow reading in children with amblyopia. (Kelly et al., 2017) Past research has shown that simulating fixation instability by jittering text during RSVP presentation does not interfere with visual acuity or crowding, but does result in slower reading speeds. (Falkenberg et al., 2007) Fixation instability may shorten the amount of time available for word recognition and decoding, thus delaying perceptual processing during reading. We did not have enough amblyopic children with good AE fixation instability (n = 6) to fully determine whether good AE stability is related to faster RSVP reading rates. Upon inspection, only 3/6 of these children had reading rates as fast as controls, with 2/3 of the faster reading children having normal FE stability.

While ocular motor dysfunction is likely involved, we cannot completely rule out other factors impacting slow reading in amblyopia. With RSVP reading, the reader cannot control their fixation duration and parafoveal preview of the letters of the next word is absent. Therefore, the visual span only needs to be large enough for the current word. The longest word tested in this study was 13 letters long (i.e., playgrounds) for the highest grade level tested (grade 6). As fixation was in the center of the word, the largest letter count to the right of fixation would have been ~6 letters. Visual span increases with age in childhood, with children in grade 6 having a visual span of 9–14 letters to the right of fixation. (Häikiö, Bertram, Hyönä, & Niemi, 2009; Rayner, 1986) Therefore, a shorter visual span is unlikely to have affected RSVP reading in our study. However, this does not necessarily rule out a role of reduced visual span in slow paragraph reading. Reduced visual span leads to an increase in forward saccades, a pattern that is present in children with amblyopia during reading. (Kelly et al., 2023; Kelly et al., 2015; Kelly et al., 2017).

Crowding can account for slow reading in amblyopia, but only when reading with the AE. (Levi et al., 2007) Crowding in the FE of individuals with amblyopia is no different than that of controls. (Kalpadakis-Smith, Tailor, Noor, & Greenwood, 2022) The FE is likely the eye that is being used to read in amblyopic children during binocular reading, especially if interocular suppression is occurring. This is supported by a studies showing slow FE reading in amblyopia. (Kelly et al., 2023; Kanonidou et al., 2010) Indeed, while the FE has normal visual acuity, it is not without its deficits. (Birch et al., 2019) For example, delayed and variable saccades and impaired motion perception are present in the FE of individuals with amblyopia. (Perdziak et al., 2016; Ho et al., 2005) FE deficits may reflect disrupted maturation of binocular neurons that respond to input from either eye. (Tao et al., 2014; El-Shamayleh et al., 2010) Impaired attention may also be a factor in slow reading in amblyopia. When shifting attention between the eyes, adults with amblyopia have trouble counting features, and attentional modulation in visual brain areas (V1, MT +) is reduced, all pointing to impaired attention in amblyopia. (Hou & Acevedo, 2021; Hou, Kim, Lai, & Verghese, 2016) However, the role of attention in slow reading remains speculative as no study has yet evaluated this factor.

## 5. Conclusions

Binocular reading in children with amblyopia is slow even when the need for inter-word saccades is removed via a RSVP task. This does not rule out a role of ocular motor dysfunction as we found slow RSVP reading to be related to FE fixation instability. Slow reading during binocular viewing may impact timed, standardized tests in later grades in children with amblyopia. Thus, it is important to continue researching the causes of slow reading in amblyopia. Our findings will inform future research investigating the causes of slow reading in children and may guide the development of reading interventions in children with amblyopia who read slowly.

## Figures and Tables

**Table 1:** Group characteristics.

	Amblyopia (n = 32)	Control (n = 30)	Amblyopia VS control
Sex: F, n (%)	18 (56)	14 (47)	*p* = 0.45
Etiology, n (%)			
Strabismus,	12 (38)	n/a	n/a
Anisometropia	11 (34)	n/a	n/a
Strabismus + anisometropia	9 (28)	n/a	n/a
Age, Mean ± SD^a^ years	10.4 ± 1.5	10.4 ± 1.4	*p* = 0.89
(range)	(8.0 to 12.5)	(8.1 to 12.9)	
Last grade completed, Mean ± SD	3.6 ± 1.5	4.0 ± 1.3	*p* = 0.31
(range)	(2 to 6)	(2 to 6)	
AE^b^ BCVA,^c^ Mean ± SD logMAR^d^	0.4 ± 0.2	0.0 ± 0.1	*p* < 0.001
(Snellen equivalent)	(20/50 ± 2 lines)	(20/20 ± 1 lines)	
(range)	(0.2 to 0.8)	(−0.2 to 0.1)	
FE^e^ BCVA, Mean ± SD logMAR	0.0 ± 0.1	0.0 ± 0.1	*p* = 0.11
(Snellen equivalent)	(20/20 ± 1 lines)	(20/20 ± 1 lines)	
(range)	(−0.2 to 0.1)	(−0.2 to 0.1)	
Stereoacuity, Mean ± SD log arcsec	3.5 ± 0.8	1.6 ± 0.1	*p* < 0.001
(range)	(1.8 to 4)	(1.5 to 1.8)	
Worth 4-dot Fusion, Mean ± SD log deg	0.3 ± 0.4	−0.2 ± 0.0	*p* < 0.001
(range)	(−0.2 to 1.2)	(−0.2 to −0.2)	

a SD, standard deviation

b AE, amblyopic eye. For control children, the right eye is listed for AE best-corrected visual acuity.

c logMAR, logarithm of the minimum angle of resolution.

d FE, fellow eye. For control children, the left eye is listed for FE best-corrected visual acuity.

## Data Availability

Data will be made available on request.

## References

[R1] Bhutada I, Skelly P, Jacobs J, Murray J, Shaikh AG, & Ghasia FF (2022). Reading difficulties in amblyopia: Consequence of visual sensory and oculomotor dysfunction. Journal of the Neurological Sciences, 442, Article 120438. 10.1016/j.jns.2022.120438 36242809

[R2] Birch EE (2013). Amblyopia and binocular vision. Progress in Retinal and Eye Research, 33, 67–84. 10.1016/j.preteyeres.2012.11.001 23201436 PMC3577063

[R3] Birch EE, & Kelly KR (2023). Amblyopia and the whole child. Progress in Retinal and Eye Research, 93, 101168. 10.1016/j.preteyeres.2023.101168 36736071 PMC9998377

[R4] Birch EE, Kelly KR, & Giaschi DE (2019). Fellow eye deficits in amblyopia. Journal of Binocular Vision and Ocular Motility., 69(3), 116–125. 10.1080/2576117X.2019.1624440 31161888 PMC6673659

[R5] Birch EE, & Stager DR (1985). Monocular acuity and stereopsis in infantile esotropia. Investigative Ophthalmology & Visual Science, 26(11), 1624–1630. 4055294

[R6] Birch EE, Subramanian V, & Weakley DR (2013). Fixation instability in anisometropic children with reduced stereopsis. Journal of AAPOS, 17, 287–290. 10.1016/j.jaapos.2013.03.011 23791411 PMC4072240

[R7] Birch E, Williams C, Drover J, (2008). Randot^®^ preschool Stereoacuity test: Normative data and validity. Journal of AAPOS., 12(1), 23–26. 10.1016/j.jaapos.2007.06.003 17720573 PMC2577836

[R8] Booth RW, & Weger UW (2013). The function of regressions in reading: Backward eye movements allow rereading. Memory & Cognition, 41(1), 82–97. 10.3758/s13421-012-0244-y 22886737

[R9] Buczkowska HA, & Kowiak BOMIS (2017). Comparison of reading speed, phonological decoding, and comprehension in the group of children with anisometropic amblyopia and control group. Optica Applicata., 47(3), 351–362. 10.5277/oa170302

[R10] Chung STL (2021). Training to improve temporal processing of letters benefits reading speed for people with central vision loss. Journal of Vision, 21(1), 14. 10.1167/JOV.21.1.14 PMC7846947 33507207

[R11] Chung STL (2011). Improving reading speed for people with central vision loss through perceptual learning. Investigative Ophthalmology & Visual Science, 52(2), 1164–1170. 10.1167/iovs.10-6034 21087972 PMC3053100

[R12] Chung STL, Kumar G, Li RW, & Levi DM (2015). Characteristics of fixational eye movements in amblyopia: Limitations on fixation stability and acuity? Vision Research, 114, 87–99. 10.1016/j.visres.2015.01.016 25668775 PMC4529398

[R13] Cotter SA, Chu RH, Chandler DL, (2003). Reliability of the electronic early treatment diabetic retinopathy study testing protocol in children 7 to <13 years old. American Journal of Ophthalmology, 136(4), 655–661. 10.1016/S0002-9394(03)00388-X 14516805

[R14] El-Shamayleh Y, Kiorpes L, Kohn A, & Movshon JA (2010). Visual motion processing by neurons in area MT of macaque monkeys with experimental amblyopia. The Journal of Neuroscience, 30(36), 12198–12209. 10.1523/JNEUROSCI.3055-10.2010 20826682 PMC2953773

[R15] Falkenberg HK, Rubin GS, & Bex PJ (2007). Acuity, crowding, reading and fixation stability. Vision Research, 47(1), 126–135. 10.1016/j.visres.2006.09.014 17078991

[R16] Ghasia FF, Otero-Millan J, & Shaikh AG (2018). Abnormal fixational eye movements in strabismus. British J Ophthalmology., 102, 253–259. 10.1371/journal.pone.0149953 28698242

[R17] Ghasia F, & Tychsen L (2024). Inter-ocular fixation instability of amblyopia: relationship to visual acuity, strabismus, nystagmus, stereopsis, vergence, and age. American Journal of Ophthalmology, 267, 230–248. 10.1016/j.ajo.2024.06.021 38944136

[R18] González EG, Wong AMF, Niechwiej-Szwedo E, Tarita-Nistor L, & Steinbach MJ (2012). Eye position stability in amblyopia and in normal binocular vision. Investigative Ophthalmology & Visual Science, 53(9), 5386–5394. 10.1167/iovs.12-9941 22789926

[R19] Häikiö T, Bertram R, Hyönä J, & Niemi P (2009). Development of the letter identity span in reading: Evidence from the eye movement moving window paradigm. Journal of Experimental Child Psychology, 102(2), 167–181. 10.1016/j.jecp.2008.04.002 18538339

[R20] Ho CS, Giaschi DE, Boden C, Dougherty R, Cline R, & Lyons C (2005). Deficient motion perception in the fellow eye of amblyopic children. Vision Research, 45(12), 1615–1627. 10.1016/j.visres.2004.12.009 15781077

[R21] Hou C, & Acevedo MG (2021). Feature counting is impaired when shifting attention between the eyes in adults with amblyopia. Frontiers in Neuroscience, 15, Article 674146. 10.3389/fnins.2021.674146 PMC8174661 34093118

[R22] Hou C, Kim YJ, Lai XJ, & Verghese P (2016). Degraded attentional modulation of cortical neural populations in strabismic amblyopia. Journal of Vision, 16(3), 16. 10.1167/16.3.16 PMC4757464 26885628

[R23] Kalpadakis-Smith AV, Tailor VK, Noor AHD, & Greenwood JA (2022). Crowding changes appearance systematically in peripheral, amblyopic, and developing vision. Journal of Vision, 22(6), 3. 10.1167/JOV.22.6.3 PMC9078053 35506917

[R24] Kanonidou E, Gottlob I, & Proudlock FA (2014). The effect of font size on reading performance in strabismic amblyopia: An eye movement investigation. Investigative Ophthalmology & Visual Science, 55(1), 451–459. 10.1167/iovs.13-13257 24370829

[R25] Kanonidou E, Proudlock F, & Gottlob I (2010). Reading strategies in mild to moderate strabismic amblyopia: An eye movement investigation. Investigative Ophthalmology & Visual Science, 51(7), 3502–3508. 10.1167/iovs.09-4236 20207968

[R26] Kelly KR, Cheng-Patel CS, Jost RM, Wang YZZ, & Birch EE (2018). Fixation instability during binocular viewing in Anisometropic and Strabismic children. Experimental Eye Research, 183, 29–37. 10.1016/j.exer.2018.07.013 30006273 PMC7323568

[R27] Kelly KR, Jost RM, De La Cruz A, & Birch EE (2015). Amblyopic children read more slowly than controls under natural, binocular reading conditions. Journal of AAPOS., 19(6), 515–520. 10.1016/j.jaapos.2015.09.002 26610788 PMC4688187

[R28] Kelly K, Jost R, De La Cruz A, (2017). Slow reading in children with anisometropic amblyopia is associated with fixation instability and increased saccades. Journal of AAPOS., 21(6), 447–451. 10.1016/j.jaapos.2017.10.001 29024763 PMC5722702

[R29] Kelly KR, Jost RM, Hudgins LA, (2023). Slow binocular reading in amblyopic children is a fellow eye deficit. Optometry and Vision Science, 100(3), 194–200. 10.1097/OPX.0000000000001995 36715973 PMC10245300

[R30] Kenyon RV, Ciujfreda KJ, & Stark L (1980). Dynamic vergence eye movements in strabismus and amblyopia: Symmetric Vergence. Investigative Ophthalmology & Visual Science, 10(1), 60–74. 7350136

[R31] Kwon M, Legge G, & Dubbels D (2007). Developmental changes in the visual span for reading. Vision Research, 47(22), 2889–2900. 10.1038/jid.2014.371 17845810 PMC2052928

[R32] Levi DM, & Klein SA (1985). Vernier acuity, crowding and amblyopia. Vision Research, 25(7), 979–991. 10.1016/0042-6989(85)90208-1 4049747

[R33] Levi DM, Song S, & Pelli DG (2007). Amblyopic reading is crowded. Journal of Vision, 7(2), 1–17. 10.1167/7.2.21 18217836

[R34] Maxwell GF, Lemij HG, & Collewijn H (1995). Conjugacy of saccades in deep amblyopia. Investigative Ophthalmology & Visual Science, 36(12), 2514–2522. 7591641

[R35] Mohney BG, & Huffaker RK (2003). Common forms of childhood exotropia. Ophthalmology, 110(11), 2093–2096. 10.1016/j.ophtha.2003.04.001 14597514

[R36] Perdziak M, Ober JK, Witkowska DK, (2016). Not only amblyopic but also dominant eye in subjects with strabismus show increased saccadic latency. Journal of Vision, 16(10), 1–11. 10.1167/16.10.12.doi 27559718

[R37] Perdziak M, Witkowska D, Gryncewicz W, Przekoracka-Krawczyk A, & Ober J (2014). The amblyopic eye in subjects with anisometropia show increased saccadic latency in the delayed saccade task. Frontiers in Integrative Neuroscience, 8, 77. 10.3389/fnint.2014.00077 25352790 PMC4196517

[R38] Rayner K (1998). Eye movements in reading and information processing: 20 years of research. Psychological Bulletin, 124(3), 372–422. 10.1037/0033-2909.124.3.372 9849112

[R39] Rayner K (1986). Eye movements and the perceptual span in beginning and skilled readers. Journal of Experimental Child Psychology, 41(2), 211–236. 10.1016/0022-0965(86)90037-8 3701249

[R40] Repka MX, Kraker RT, Beck RW, (2008). Monocular oral reading performance following amblyopia treatment in children. American Journal of Ophthalmology, 146(6), 942–947. 10.1016/j.ajo.2008.06.022 18708179 PMC2713113

[R41] Rosenbaum A, Santiago A. Clinical Strabismus Management. (Rosenbaum A, Santiago A, eds.). WB Saunders; 1999.

[R42] Shaikh AG, Otero-Millan J, Kumar P, & Ghasia FF (2016). Abnormal fixational eye movements in amblyopia. PLoS One, 11(3), e0149953. 10.1371/journal.pone.0149953 26930079 PMC4773232

[R43] Stifter E, Burggasser G, Hirmann E, Thaler A, & Radner W (2005). Evaluating reading acuity and speed in children with microstrabismic amblyopia using a standardized reading chart system. Graefe’s Archive for Clinical and Experimental Ophthalmology, 243(12), 1228–1235. 10.1007/s00417-005-1187-9 16003512

[R44] Stifter E, Burggasser G, Hirmann E, Thaler A, & Radner W (2005). Monocular and binocular reading performance in children with microstrabismic amblyopia. The British Journal of Ophthalmology, 89(10), 1324–1329. 10.1136/bjo.2005.066688 16170125 PMC1772895

[R45] Subramanian V, Jost RM, & Birch EE (2013). A quantitative study of fixation stability in amblyopia. Investigative Ophthalmology & Visual Science, 54(3), 1998–2003. 10.1167/iovs.12-11054 23372053 PMC3604910

[R46] Tao X, Zhang B, Shen G, (2014). Early monocular defocus disrupts the normal development of receptive-field structure in V2 neurons of macaque monkeys. The Journal of Neuroscience, 34(41), 13840–13854. 10.1523/JNEUROSCI.1992-14.2014 25297110 PMC4188977

[R47] Tarczy-Hornoch K, Varma R, Cotter SA, (2011). Risk factors for decreased visual acuity in preschool children: The multi-ethnic pediatric eye disease and baltimore pediatric eye disease studies. Ophthalmology, 118(11), 2262–2273. 10.1016/j.ophtha.2011.06.033 21856014 PMC3208077

[R48] Tarita-Nistor L, Brent MH, Steinbach MJ, Markowitz SN, & González EG (2014). Reading training with threshold stimuli in people with central vision loss: A feasibility study. Optometry and Vision Science., 91(1), 86–96. 10.1097/OPX.0000000000000108 24212184

[R49] Webber AL, Mandall TR, Molloy DT, Lister LJ, & Birch EE (2020). Worth 4 dot app for determining size and depth of suppression. Translational Vision Science & Technology, 9(4), 1–11. 10.1167/tvst.9.4.9 PMC7396169 32818097

